# Saving costs in cancer patient management through molecular imaging

**DOI:** 10.1007/s00259-017-3804-3

**Published:** 2017-08-14

**Authors:** Carl von Gall, Piotr Maniawski, Fred Verzijlbergen, Ignasi Carrio, Thomas Beyer, Antonis Kalemis

**Affiliations:** 1 Siemens Healthineers, Molecular Imaging, Knoxville, TN USA; 2 Philips Healthcare, Advanced Molecular Imaging, Cleveland, OH USA; 3 Department of Nuclear Medicine, Radboud University Medical Centre, Nijmegen, The Netherlands; 4 Department of Nuclear Medicine, Hospital de la Santa Creu i Sant Pau, Barcelona, Spain; 5 QIMP Group, Center for Medical Physics and Biomedical Engineering, General Hospital Vienna, Medical University of Vienna, 4L Waehringer Guertel 18-20, A-1090 Vienna, Austria; 6 European Industrial Association for Nuclear Medicine and Molecular Healthcare (AIPES eeig), Brussels, Belgium; 7 Siemens Healthineers, Magnetic Resonance Imaging, Erlangen, Germany

Precision medicine is the future of clinical medicine where one-size-fits-all healthcare guidelines are refined to fit the needs of specific patient groups and even individuals, using molecular, genetic, proteomic and/or other types of data acquired from different diagnostic procedures at different stages of the disease. The ultimate goal is to anticipate and prevent disease in the healthy and to precisely diagnose and treat disease in the ill. However, the success of precision medicine depends on having accurate tests that identify patients who can benefit from expensive, targeted therapies [1]. One of the prominent tools of personalised medicine is molecular imaging, which has the potential to drive tomorrow’s healthcare by lowering healthcare costs and improving health outcomes.

The possible benefits of molecular imaging include the improvement of definitive diagnoses, treatment planning, targeted therapy selection, early treatment response assessment, treatment follow-up, and drug development. Its successful and comprehensive role in managing cancer has also made the molecular imaging industry an important sector of Europe’s economy. According to data from the Association of Imaging Producers & Equipment Suppliers (AIPES - http://www.aipes-eeig.org/), the business sector of nuclear medicine and molecular imaging now generates more than EUR 1.25 billion in annual revenue within Europe. Nevertheless, the molecular imaging industry still faces significant barriers to its wider clinical adoption throughout Europe. In particular, easier regulatory processes and market access frameworks are needed before molecular imaging can demonstrate its full capabilities.

## Clinical evidence

Extensive clinical evidence has established the effectiveness and value of molecular imaging for cancer diagnosis and management [2]. Technologies are deemed a good value when their additional costs, if any, are acceptable when compared to their ability to improve health outcomes, as measured by increased longevity and/or better quality of life for patients. For instance, an intervention’s impact on health outcomes is often represented by a standardised outcome measure called the quality-adjusted life-year (QALY), which provides a single-number summary of how the intervention affects both survival (in life years) and quality of life. Other standardised outcome measures include the disability-adjusted life year (DALY), life-year gained (LGY), and health utility index (HUI). These measurements of health outcomes are compared to the lifetime costs of the intervention when assessing its value. Using this methodology, studies on the cost effectiveness of molecular imaging over the last 20 years have clearly demonstrated its value for several cancer indications, as well as cancer drug development [2].

One of the most widely adopted applications of molecular imaging is the use of PET/CT for preoperative staging of both non-small-cell lung cancer (NSCLC) and solitary pulmonary nodules. Clinical trials and cost-effectiveness analyses together have established that PET/CT provides accurate preoperative staging of lung cancer [3], resulting in a decrease in unnecessary surgeries and treatment costs [2]. For example, cost-effectiveness analyses performed alongside a randomised clinical trial showed that preoperative staging with PET/CT reduced the number of futile thoracotomies by almost 20% for patients with highly suspected NSCLC — saving an average of EUR 19,314 for each futile thoracotomy that was avoided and significantly improving the patients’ quality of life [4]. Furthermore, studies on the cost-effectiveness of PET and PET/CT in the diagnosis of solitary pulmonary nodules concluded that it was cost effective, particularly for patients with a low or high probability of malignancy in which each additional QALY cost as little as USD 20,000 or USD 16,000, respectively [5]. In addition, studies have found that PET/CT significantly improves radiotherapy treatment planning for patients with lung cancer by providing more precise target delineation, so a higher dose can be delivered to a smaller volume [6].

PET/CT also, cost-effectively, provides definitive diagnoses and staging for colorectal cancer [2]. Clinical trials have shown that PET/CT precisely localises and characterises malignant colorectal lesions, improving the overall accuracy of staging and restaging [7]. In particular, the liver is the most common site of recurrence for patients treated for colorectal cancer. When liver metastases occur, it is critical to identify the number and location of the metastases to determine whether the patient is a good candidate for liver resection. Adding PET/CT to the diagnostic pathway more accurately selects the patients that can benefit from surgery. An economic analysis determined that using PET in the diagnosis and staging of colorectal cancer patients with liver metastases saved on average of EUR 2671 per patient with the same average life expectancy as using CT alone, while also avoiding unnecessary exploratory surgeries for 6.1% of the patients [8]. Furthermore, a clinical trial showed that the additional cost of adding PET to treatment planning for patients with potentially resectable colorectal liver metastases was compensated by a 38% reduction in futile laparotomies compared with a conventional diagnostic workup, providing a net monetary benefit ranging from EUR 1004 to EUR 11,060 depending on the monetary value given to a QALY [9].

Studies also show that it is cost effective to use PET/CT to diagnose, stage, and plan treatments for patients with head and neck cancer [2]. Chemotherapy is the standard of care for patients with head and neck cancer, but it is not highly effective for nodal disease. Hence, it is common to surgically remove potentially cancerous lymph nodes as an adjunct to chemotherapy. Adding PET/CT to the diagnostic pathway more accurately selects patients who need neck dissection. Studies have found that patients receiving PET/CT surveillance had the same probability of survival or better, while avoiding unnecessary surgeries and potential complications. For example, a randomised, controlled trial for squamous cell carcinoma therapy follow-up found that FDG PET/CT surveillance resulted in “*considerably fewer operations and it was more cost-effective*” than planned neck dissections. Specifically, the number of patients receiving neck dissections was reduced from 78% to 19% with the use of PET/CT, lowering the cost per patient on average by EUR 1492 during the duration of the study [10].

Molecular imaging is also a powerful way to identify and localise primary and recurrent prostate cancer with the emergence of promising new radiotracers, such as [^11^C]choline, [^18^F]fluorocholine, [^11^C]acetate, [^18^F]FACBC, and agents targeting prostate-specific membrane antigen (PSMA) [11–13]. Prostate cancer is a biologically and clinically heterogeneous disease that ranges from indolent to aggressive forms, and PET/CT enables a cost-effective guidance of therapy selection and management of prostate cancer. As such, a PET/CT scan can identify whether there are metastases to help determine if the patient is a suitable candidate for surgery, or if he needs salvage radiotherapy to the prostate bed, thus improving therapy selection and reducing treatment side effects. PET/CT imaging can also play a critical role in treatment monitoring, quickly identifying which patients are responding to treatment on a value-based model. Finally, emerging prostate-specific membrane antigen-targeting theranostic agents for PET and SPECT, such as [^177^Lu]PSMA-617 or [^177^Lu]J591, are demonstrating outstanding diagnostic and targeted radiotherapeutic capabilities for patients with metastatic castration-resistant prostate cancer [14, 15].

The use of PET/CT also provides cost-effective definitive diagnosis, staging, targeted therapy assessment, and treatment follow-up for both Hodgkin and non-Hodgkin lymphoma [16, 17]. For example, the International Conference on Malignant Lymphomas recommends that PET/CT (instead of CT alone) should be the standard of care for mid-treatment assessment and remission assessment for patients with Hodgkin lymphoma, diffuse large B-cell lymphoma, and aggressive follicular lymphoma [15]. In addition, cost-effectiveness analyses have shown a 96% accuracy in restaging patients with Hodgkin lymphoma with a net cost savings of USD 3268 for each correctly detected case [18].

Last but not least, PET/CT can be used to expedite and reduce the cost of developing new cancer drugs by identifying which experimental drugs are likely to fail at a much earlier stage [19–21]; clinical trials of ineffective drugs can then be stopped more quickly and limited resources can be redirected to more promising drugs under development. The drug development process has low success rates at every stage, leading to a typical cost of USD 1.9 billion for each newly approved drug [20]. Molecular imaging applied in the initial stages of drug development can lower this cost by providing evidence of drug biodistribution, pharmacodynamic changes, on-target drug effects, and surrogate efficacy endpoints, as well as by identifying patients who are more likely to benefit from the drug treatment. As a result, “precision pharmacology” can lead to well-designed, smarter clinical trials that answer key questions earlier and improve decision-making.

Overall, extensive clinical evidence demonstrates that molecular imaging is both cost effective and critical for cancer diagnosis and management. In particular, it helps select the most appropriate cancer treatment for each patient so ineffective therapies can be avoided or quickly discontinued, which reduces the patients’ costs, side effects, and emotional burden.

## Challenges and recommendations

Despite the proven clinical benefits of using molecular imaging for oncology applications, some clinicians have been slow to adopt this technology due to a few significant barriers. However, there are concrete steps that can be taken to overcome these barriers, and it is critical to do so to reach the dream of personalised medicine.

There are many promising new personalised treatments, including those based on immunotherapy, genomics, and proteomics. Unfortunately, these therapies are currently very expensive and seem to work in only about 10–20% of the target population. Radiolabelling these new drugs can help select the right patient for the right drug with the right dose. Molecular imaging can act as a “gatekeeper” — identifying whether there is overall high expression in all lesions, thus, assisting in the justification of the choice of a highly-specific, personalised treatment, in light of its expense or potential side effects. For example, there are personalised treatments for breast cancer that target the overexpression of the HER2 receptor on the cell membrane, which occurs in approximately 30% of human breast tumours and is associated with significantly worse prognosis in patients with node-positive breast cancer [22]. Drugs like monoclonal antibody trastuzumab that target HER2 receptor positive breast cancer cost approximately EUR 40,000 to EUR 50,000 per cycle; whereas, their effectiveness for a specific patient is unclear given pathology results that are typically only available for one or two biopsied lesions rather than all lesions and metastases [23, 24]. Molecular imaging can help solve this problem by identifying patients that will benefit from such highly specific, costly treatments.

In order to reach this potential, changes need to be made to the regulatory processes. Radiopharmaceuticals are given in trace amounts (pmol to μmol) and side effects are rare. Analysing data from over one million radiopharmaceutical administrations, Silberstein demonstrated that “*the incidence of adverse effects has remained stable at 2.1–2.3/10* ^*5*^ *dosages*” [25]. Despite these facts, radiopharmaceuticals are currently regulated as full pharmaceuticals. Treating these compounds as ordinary drugs inhibit bringing innovative new radiopharmaceuticals to the clinic and market. We need a pathway to easier commercialization for the most critical compounds. For example, the lymphoma imaging drug [^99m^Tc]-Rituxmab is an approved radionuclide attached to an already approved drug that has been licenced and is in clinical use for therapies at much higher doses, replacing the ^99m^Tc with beta-emitters, accordingly. Despite this, safety and efficacy proofs are still required for the use of trace amounts of this radiopharmaceutical before it can be used to guide very expensive antibody treatments, which have a probability of success of only 10–20% [26, 27]. [^99m^Tc]-Rituximab, in particular, is a good example of the amount of time needed to receive regulatory clearance and reimbursement, as well as, the role this delay had in destroying the business case for its commercialisation. As a result, radiolabelled rituximab never reached the clinic, as alternative treatment options received faster approval. Imaging agents, such as [^99m^Tc]-Rituximab should not have the same regulatory requirements as full pharmaceuticals.

Assessing the rapidly evolving molecular imaging technologies is very difficult, but these new technologies lead to key improvements in radiotracers, image quality, quantification, and lower radiation doses. Quantification is particularly important as we move towards population health using big data and deep learning algorithms. Unfortunately, large, multi-centre, randomised clinical trials are too slow and costly to effectively evaluate these rapidly changing technologies. By the time a technology is fully assessed with today’s full-fledged clinical trial requirements, it is often already succeeded by newer methodologies. Therefore, small, smart trials for rapid marketing authorization are essential in order to allow rapid dissemination of such technological innovation in the clinic, particularly when significant evidence on safety is already available. Rather than setting all key trial parameters at the start of a clinical trial, adaptive trials are needed that make planned, well-defined changes in these parameters during the trial based on the acquired data. For instance, Bayesian adaptive approaches could be used to incorporate knowledge of signal along the path of data collection [28]. Such adaptive clinical trials can improve efficiency, reduce cost, maximise the information obtained, and minimise risk to the subjects and sponsor. However, these studies require novel funding schemes and platforms from both industry and governmental agencies — funding should not stop with discovery. The suggestions above are prerequisites for proving the benefits of molecular imaging in a straightforward and fast manner.

When appropriate, value-based approaches, such as health cost-benefit analyses, could also replace clinical trials, as they are faster and more cost-effective than the latter. For example, cost-benefit analyses could be used when assessing PET/CT, SPECT/CT or PET/MR for an indirect clinical benefit. Namely, molecular imaging provides an indirect benefit when it replaces another imaging modality and provides both improved diagnostic accuracy and changes in patient management that lead to improved health outcomes. This initial assessment should be performed using decision modelling. If these analyses are inconclusive, then further randomised clinical trials could be performed, and they should focus from the onset on whether molecular imaging improves health outcomes such as quality of life.

Overall, there needs to be an acknowledgement that waiting for conclusive, standard clinical trial results has also a cost in terms of mortality, quality of life, and financial savings. The healthcare industry needs to make informed decisions based on the best available data at the time. If there is a low chance of patient harm and a high probability of patient benefit and/or savings, then the use of molecular imaging should be approved.

However, both modelling and smart clinical trials are still done for a specific healthcare environment with specific treatment patterns, tariffs, costs, and reimbursement policies. These specifics vary significantly between countries, even in regions such as Europe, where despite the overall unification efforts, the national regulatory and reimbursement mechanisms still remain substantially different. To address this issue, different actions are needed at several levels. At the hospital level, some degree of standardisation is essential in order to acquire comparable data that can be pooled together into centralised registries or other similar initiatives that would facilitate larger scale studies.
